# A Multifunctional Frontloading Approach for Repeated Recycling of a Pressure-Controlled AFM Micropipette

**DOI:** 10.1371/journal.pone.0144157

**Published:** 2015-12-04

**Authors:** Phillip Roder, Carsten Hille

**Affiliations:** Department of Physical Chemistry / Applied Laser Sensing in Complex Biosystems (ALS ComBi), Institute of Chemistry, University of Potsdam, Potsdam, Germany; University of Bern, SWITZERLAND

## Abstract

Fluid force microscopy combines the positional accuracy and force sensitivity of an atomic force microscope (AFM) with nanofluidics *via* a microchanneled cantilever. However, adequate loading and cleaning procedures for such AFM micropipettes are required for various application situations. Here, a new frontloading procedure is described for an AFM micropipette functioning as a force- and pressure-controlled microscale liquid dispenser. This frontloading procedure seems especially attractive when using target substances featuring high costs or low available amounts. Here, the AFM micropipette could be filled from the tip side with liquid from a previously applied droplet with a volume of only a few μL using a short low-pressure pulse. The liquid-loaded AFM micropipettes could be then applied for experiments in air or liquid environments. AFM micropipette frontloading was evaluated with the well-known organic fluorescent dye rhodamine 6G and the AlexaFluor647-labeled antibody goat anti-rat IgG as an example of a larger biological compound. After micropipette usage, specific cleaning procedures were tested. Furthermore, a storage method is described, at which the AFM micropipettes could be stored for a few hours up to several days without drying out or clogging of the microchannel. In summary, the rapid, versatile and cost-efficient frontloading and cleaning procedure for the repeated usage of a single AFM micropipette is beneficial for various application situations from specific surface modifications through to local manipulation of living cells, and provides a simplified and faster handling for already known experiments with fluid force microscopy.

## Introduction

Microchanneled atomic force microscopy (AFM) micropipettes are a versatile nanodispensing (NADIS) system, which can deliver the smallest necessary volumes and has facilitated many applications in surface functionalization [1,2], adhesion [3,4], spatial cell manipulation [5–7], injection [5,8] and lithography/nanoprinting [9] in recent years. In the first NADIS experiments, the dispensing of liquid was limited by capillarity and the opening of the tip [10,11]. Due to combination with an external pump and a special probe holder, small volumes in *fL*- up to the *aL*-range could be delivered in a pressure- and time-dependent manner both in air and liquid environments [5,7,9]. The combination of conventional AFM and nanofluidics (fluidic force microscopy (FluidFM)) makes this method attractive for biological applications in aqueous environments. Compared to conventional glass pipettes, this tool is particularly suitable when using substances of high cost or limited amounts, because significantly less volume is required for an experiment [12,13]. Another advantage over glass pipettes is the precise control wielded in the manipulation of sensitive targets due to concurrent measurements of cantilever deflections without significant target damage [5]. Thus, targets such as functionalized surfaces or biological tissues can be precisely and gently manipulated physically, biologically and chemically with the smallest pipette in the world [6,14–16].

In order to recycle these micropipettes several times, a specific loading method and cleaning process are required which can be applied rapidly and effectively for various application situations. The loading procedure of an AFM micropipette, which is often described in the literature [7], is carried out *via* the reservoir (backloading). In this case, approx. 10 μL of liquid is applied to the reservoir and the liquid is subsequently pressed with a given high pressure through the microchannel up to the tip opening of the micropipette. One major disadvantage of this loading procedure is the required bypassing of the dead volume (approx. 0.5 μL volume of the microchannel between the reservoir and the tip opening of the micropipette). Due to very low volumetric flow rates (e.g. ~60 fL mbar^-1^ s^-1^ using a tip opening of 8 μm [7]), it normally takes between several hours and half a day (for smaller tip openings) to totally exchange a previously loaded liquid volume with another liquid. Although the time-consuming backloading procedure is possible, a subsequent cleaning procedure of such a loaded micropipette seems to also be very time-consuming, and complete removal of the liquid from the entirety of the microchannel is not guaranteed. For this reason, the backloading procedure is suitable when using only one target substance per micropipette. However, many experiments require several different or freshly prepared substances (e.g. fast surface functionalization or physiological/pharmacological experiments in living tissues) [17]. Thus, frontloading seems to be a more suitable method which is easy to handle and less time-consuming, as shown below. The aim of this study was to develop a quick and operative frontloading method for a micropipette with an optimized cleaning procedure for different target solutions (e.g. fluorescent dyes, proteins). The results presented here are essential for versatile surface functionalization and cell manipulation in cases where several different substances use the same AFM micropipette.

## Material and Methods

### Combined FluidFM and fluorescence microscope setup

In the present study, we used a FlexAFM scan head (Nanosurf, Langen, Germany), which was combined with a microchanneled AFM micropipette (Cytosurge, Zurich, Switzerland) *via* a probe holder. Control of the scan head and the nanofluidics was ensured by the Nanosurf C3000 controller (Nanosurf, Langen, Germany) and the CytosurgeUI software ver. 1.0.41 (Cytosurge, Zurich, Switzerland). The flat AFM tip consisted of silicon nitride, a reflective gold-chrome coating, and featured a length of 200 μm, a width of 36 μm and a tip opening of 2 μm. The micropipette was connected to a pressure controller *via* a pneumatic connector and a silicone tube. Thus, pressure could be applied between -800 mbar (low pressure) and 1125 mbar (high pressure). The height position or, rather, deflection of the cantilever relative to the target surface, could be controlled by a standard near infrared AFM laser detection system [3]. For the acquisition of bright-field images and corresponding fluorescence images, the AFM scan head was mounted on a specifically adapted AFM sample stage (Nanosurf, Langen, Germany), which could be connected to a Zeiss Axio Observer Z1 inverted microscope (Carl Zeiss, Jena, Germany). The microscope was equipped with a Zeiss Fluar 20×/NA 0.75 objective and two different filter cubes for separation of excitation and emission light at λ_ex_ = 485 nm ± 15 nm and λ_em_ = 535 nm ± 25 nm or at λ_ex_ = 640 nm ± 15 nm and λ_em_ = 700 nm ± 37.5 nm. After fluorescence excitation with an X-Cite short-arc lamp (Visitron Systems, Puchheim, Germany), image acquisition occurred with a CoolSnap HQ^2^ CCD camera (Photometrics, Arizona, USA) using the software MetaMorph ver. 7.1 (Molecular Devices, Sunnyvale, USA). The image analysis and graphical presentation were performed with CorelDRAW X3 (Corel, Munich, Germany), ImageJ ver. 1.49n and Origin 9.1G (Origin Lab Corp., Northampton, USA).

### Frontloading and cleaning procedure

**Fig 1:**
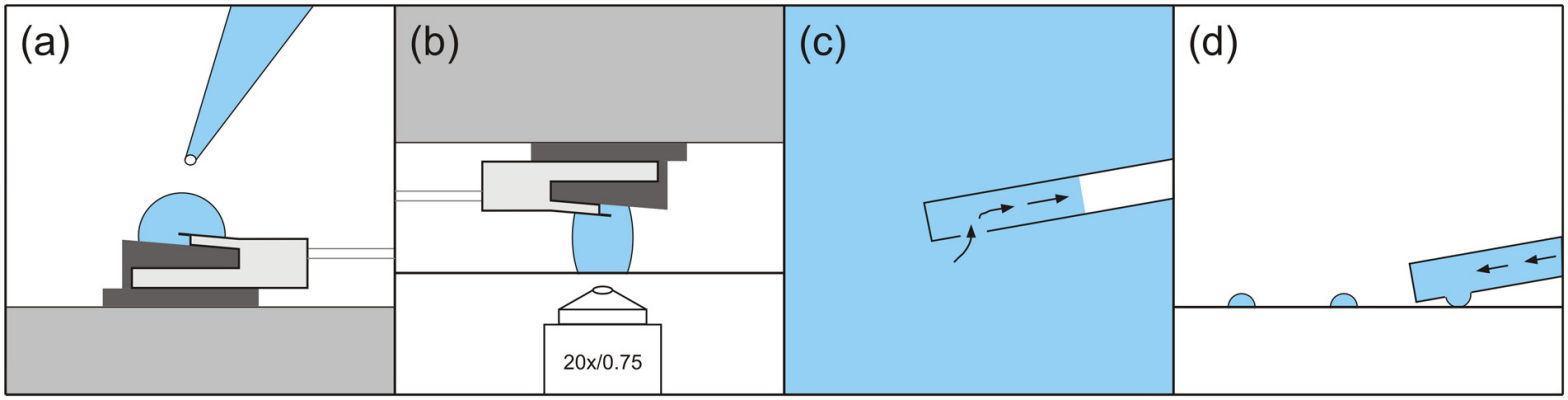
Representation of the AFM micropipette frontloading procedure. Schematic illustration of (a) volume delivery on top of the probe holder by using a pipette, (b) loading position of the AFM scan head in the liquid above a chucked coverslip for frontloading (the lamellar liquid film reduces contamination of the entire clip), (c) zoom-in of (b) illustrating the frontloading process at the micropipette opening using low pressure (the black arrows indicate the running direction of the liquid), (d) dispensing of small liquid droplets onto a glass coverslip surface after successful frontloading procedure.

To realize the frontloading procedure, a volume of about 5–50 μL containing the target substance was initially applied on top of the micropipette (Fig 1a) or directly on a glass coverslip surface. The AFM probe was then placed above the coverslip in such a way that the droplet was in contact with both, obtaining only low contamination of the micropipette with the liquid (Fig 1b). Then, a low pressure of approx. -700 mbar was applied for a few seconds and the micropipette could be filled with the solution (Fig 1c). After this loading process, the residual liquid adhering to the micropipette or glass coverslip was removed using an Eppendorf pipette so that it could be reused for other experiments. Although minimization of the applied droplet volume is already possible down to approx. 5–10 μL, thus minimizing the lost volume as well, the handling of such small volumes with the AFM probe would still be rather difficult. Then, the AFM scan head was positioned above the target surface. Subsequently, the micropipette could be moved with a predetermined setpoint to the target surface (e.g. surface of a coverslip) and a droplet could be placed on the surface by means of a short high-pressure pulse (Fig 1d).

For external cleaning of the micropipette, a large drop of 4% NaOCl solution (Carl Roth, Karlsruhe, Germany) was added onto the micropipette surface. After a few seconds (approx. 20–60 s) of incubation, the micropipette was rinsed with bidistilled water. For internal cleaning of the microchannel, the micropipette tip was dipped into a specific cleaning droplet. Water and ethanolic cleaning solutions are well suited for small polar target substances (e.g. organic fluorophores, neurotransmitters), whereas NaOCl solutions are suited for proteins and other larger biological compounds (e.g. antibodies). Through repeated loading (low pressure -700 mbar) and release (high pressure 1125 mbar) cycles, the micropipette microchannel could be rinsed. The number of rinsing cycles depends on the previously used liquid (e.g. concentration, interaction with pipette surface) and the applied cleaning solution. After this cleaning process, the micropipette could tentatively be loaded again with bidistilled water and a dispensed water droplet was therefore checked for residual contamination, e.g. by fluorescence measurements. The usual cleaning period for exchanging one target solution with another required approx. 45 min. All target and cleaning solutions were filtered and degassed in order to prevent plugging of the microchannel due to residual solid matter or air bubbles.

### Target substances

To demonstrate the feasibility of the suggested frontloading and cleaning procedures, two types of target substances were tested, namely the organic fluorescent dye rhodamine 6G (Sirah Lasertechnik, Grevenbroich, Germany) and as an example of a larger biological compound, the secondary antibody goat anti-rat IgG labeled with the dye AlexaFluor647 (Life Technologies, Darmstadt, Germany). The target substances were dissolved in bidistilled water or phosphate buffered saline (Sigma-Aldrich, Taufkirchen, Germany) and diluted to final concentrations of 50 μM rhodamine 6G and 10 μg/mL secondary antibody.

## Results and Discussion

### Frontloading and controlled delivery of an organic fluorescent dye with subsequent cleaning

**Fig 2:**
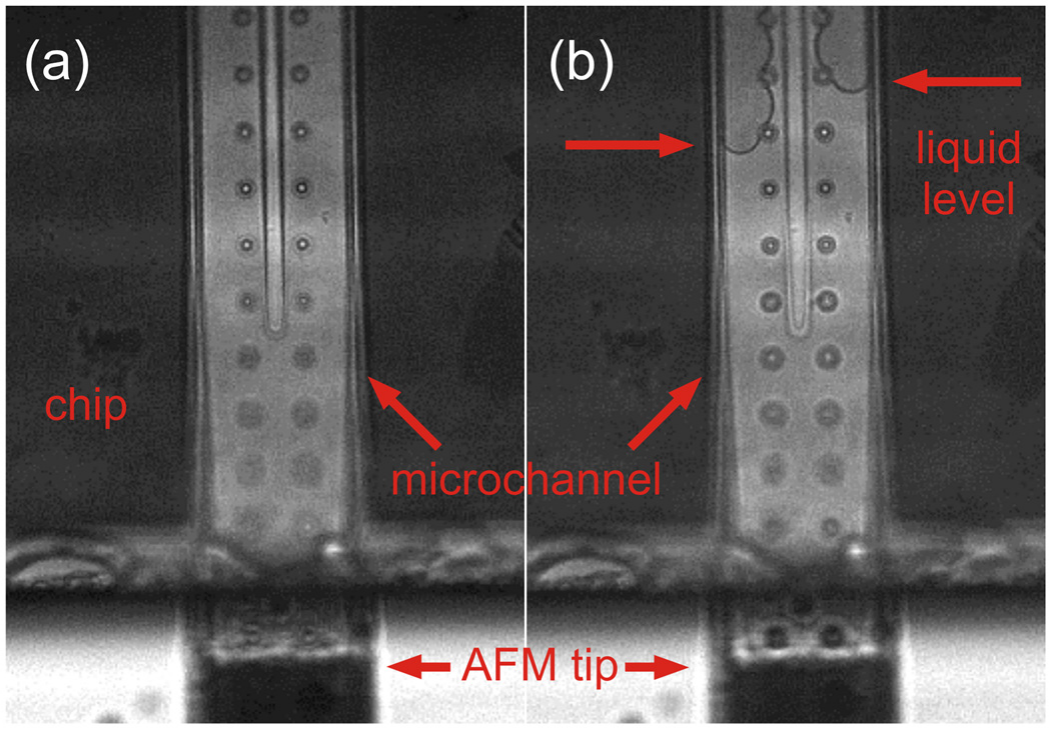
Visualization of the AFM micropipette liquid level. Bright-field images of a micropipette microchannel (a) before and (b) after frontloading procedure resulting in a stable liquid level.

**Fig 3:**
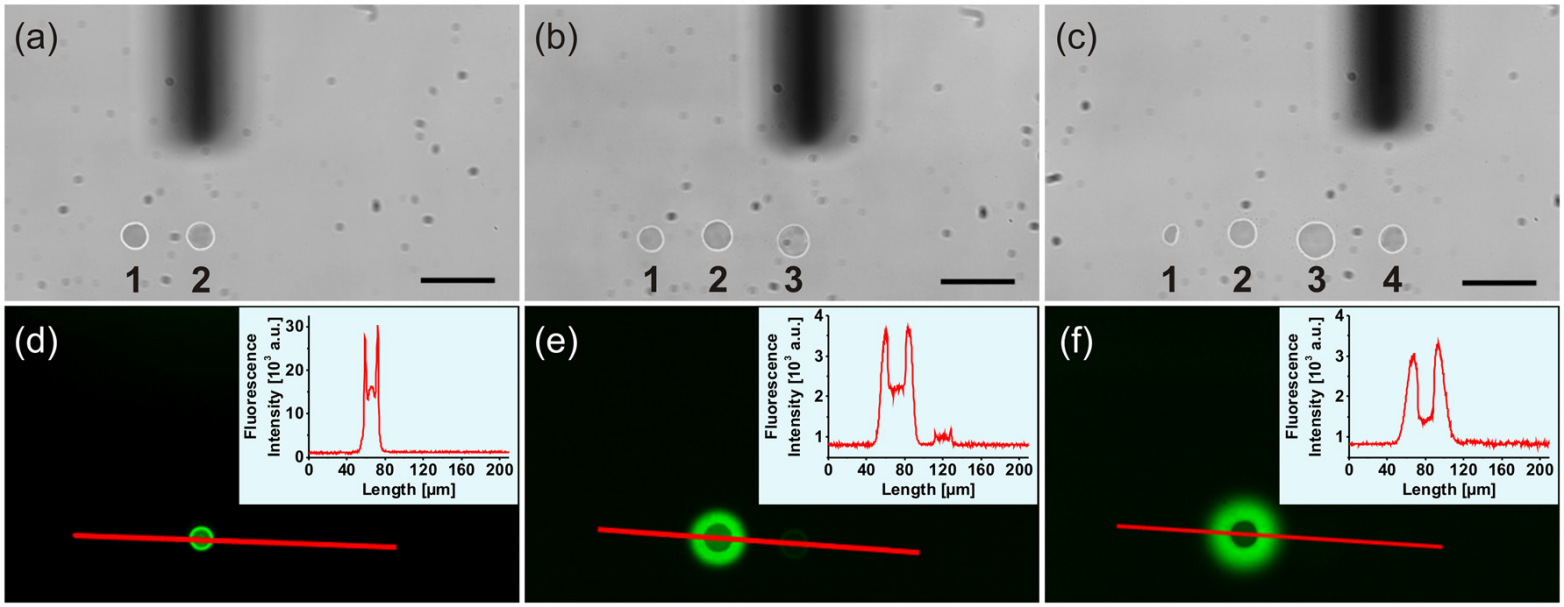
AFM micropipette cleaning procedure when using the organic dye rhodamine 6G. (a-c) Bright-field images of four sequentially spotted droplets (pos. 1, 3 and 4: glycerol-water; pos. 2: 50 μM rhodamine 6G); micropipette silhouette can be seen in the background; scale bar = 50 μm. (d-f) Corresponding fluorescence images of the identical regions shown in (a-c) with associated intensity profile plots along the red lines; λ_ex_ = 485 nm ± 15 nm, λ_em_ = 535 nm ± 25 nm; time periods between the delivery of the individual droplets: 1 to 2 in 12 min, 1 to 3 in 39 min, 1 to 4 in 70 min.

Rhodamine 6G and its derivatives are organic dyes frequently used in fluorescence microscopy because of their high photostability and favorable excitation and emission properties in the visible spectral range. Thus, we tested the frontloading of the AFM micropipette with 50 μM rhodamine 6G diluted in water and then analyzed the required cleaning cycles of the micropipette by recording the residual fluorescence. For frontloading, a droplet of 50 μM rhodamine 6G was placed on top of the probe holder and the micropipette was then loaded, applying -700 mbar low pressure for only 4 s. By using the microscopic view, it could be observed that the suctioned liquid was immediately pressed out of the microchannel by the air column already existing in the microchannel. It is highly probable that after frontloading a higher pressure existed inside the microchannel than outside. However, by adding 20 wt% glycerol to the aqueous rhodamine 6G solution, the extrusion of the solution could be prevented, and in addition, the droplets did not evaporate rapidly after delivery onto a surface. Reasons for this behavior can be found in the higher viscosity, lower vapor pressure and changed surface properties such as hydrophobicity [18–21]. Indeed, frontloading of the micropipette with a glycerol-containing solution resulted in a stable liquid level in the microchannel (Fig 2). Afterwards, a droplet of rhodamine 6G was delivered onto a glass coverslip and the fluorescence was detected (Fig 3a and 3d, pos. 2).

For external cleaning, a drop of 4% NaOCl was placed on top of the probe holder and removed after about 20 s. The micropipette was then dipped into a 5% ethanol-water solution and the microchannel was rinsed five times (each for 5 s) by alternate applications of low pressure (-700 mbar) and a high pressure (1125 mbar) using the external Cytosurge pressure controller software. Thereafter, the micropipette was completely emptied and filled with a 20 wt% glycerol-water solution, applying a low pressure of -700 mbar for 4 s. Then, a glycerol-water droplet could be delivered onto the glass surface next to the previously released rhodamine 6G droplet (Fig 3b, pos. 3). Despite the first cleaning procedure, a low residual fluorescence signal could still be detected in the glycerol-water droplet (Fig 3e). For this reason, the micropipette was cleaned once again as described above and a second droplet of glycerol-water was placed on the glass surface (Fig 3c, pos. 4). At that point, no residual fluorescence signal could be observed when using the same experimental image acquisition conditions (Fig 3f). In addition, the disappearance of the low fluorescence signal from the third droplet primarily shown in Fig 3e was most likely the result of a drying effect. However, due to the lack of a fluorescence signal in the freshly delivered fourth droplet, it could be assumed that the micropipette was sufficiently cleansed of rhodamine 6G after the two cleaning steps.

**Fig 4:**
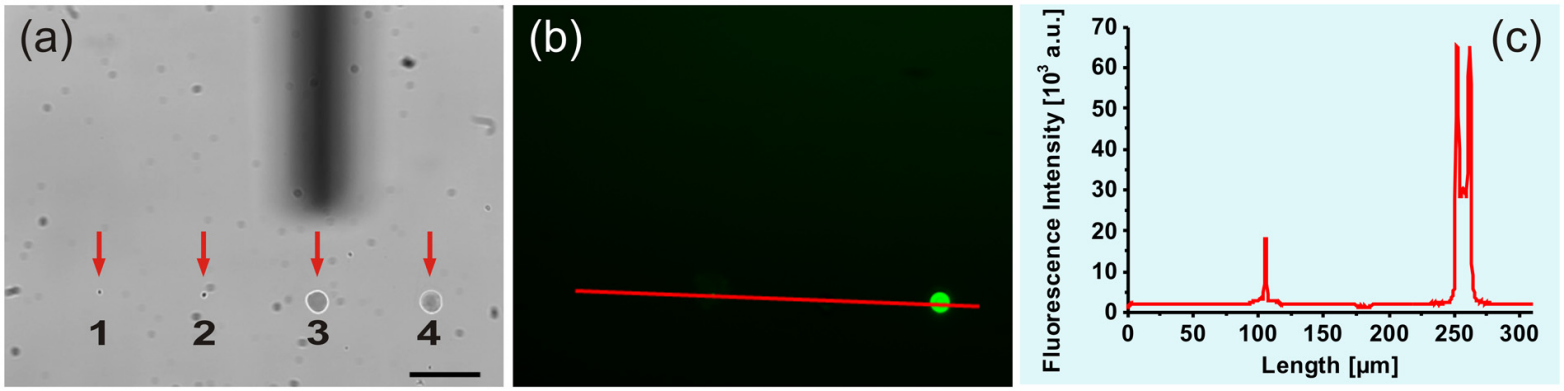
Alternate spotting of water and rhodamine 6G. (a) Bright-field image of four sequentially spotted droplets within 100 min (pos. 1 and 3: glycerol-water; pos. 2 and 4: 50 μM rhodamine 6G); the silhouette of the micropipette can be seen in the background; scale bar = 50 μm; time periods between the delivery of the individual droplets: 1 to 2 in 27 min, 1 to 3 in 74 min, 1 to 4 in 100 min. (b) Corresponding fluorescence image; λ_ex_ = 485 nm ± 15 nm, λ_em_ = 535 nm ± 25 nm. (c) Fluorescence intensity profile plot along the red line indicated in (b).

With this established frontloading and cleaning procedure, rhodamine 6G and glycerol-water droplets could be delivered sequentially onto a glass surface using the same micropipette. In Fig 4a, a bright-field image of four sequentially spotted liquid droplets is shown, in which the first and third droplet contained glycerol-water and the second and fourth droplets contained rhodamine 6G. As shown in the corresponding fluorescence image in Fig 4b, only the rhodamine 6G-droplets exhibited a fluorescence signal, as expected. However, the fluorescence intensities from these droplets differed significantly (Fig 4c). The reason for this is probably the time-dependent drying-out of the spotted dye droplets and thereby the appearance of changed fluorescence properties (compare also the spot sizes in Fig 4a). This evaporation effect is particularly strong for small *fL*-volumes, because such droplets exhibit larger surface-to-volume ratios in comparison to droplets of *mL*-volumes. By using a sensitive mass sensor, previous studies showed that evaporation of such small droplets occurred within only a few minutes or even faster [18]. Indeed, the acquisition of a time series of a delivered rhodamine 6G droplet prompted a decrease in the fluorescence intensity as well as a change in the droplet shape during drying within 60 min (S1 Fig). This time-dependent trend confirmed the previously observed changes from droplet 2 to droplet 4 in Fig 4c.

### Frontloading and controlled delivery of a biological compound and micropipette storage

**Fig 5:**
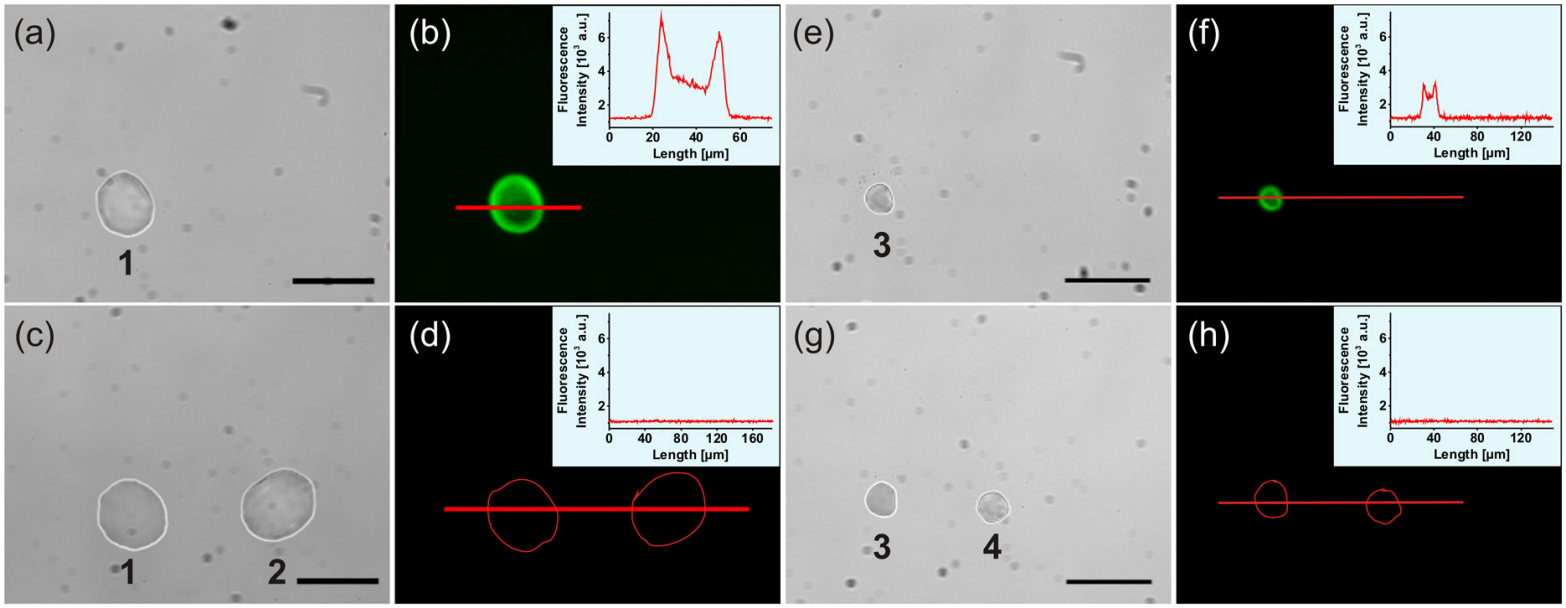
Repeated AFM micropipette usage for antibody spotting. (a) Bright-field image of a spotted droplet of AlexaFluor647-labeled antibody IgG (10 μg/mL) and (b) the corresponding fluorescence image with an intensity profile plot; scale bar = 50 μm; λ_ex_ = 640 nm ± 15 nm, λ_em_ = 700 nm ± 37.5 nm. (c,d) Bright-field image and corresponding fluorescence image of the same spot as shown in (a,b, pos. 1), as well as the subsequently spotted glycerol-water droplet after the cleaning procedure (pos. 2); time period between the delivery of droplets 1 and 2 about 65 min. (e,f) Bright-field image and corresponding fluorescence image with an intensity plot of a spotted antibody droplet (pos. 3) due to repeated frontloading of the same micropipette after several hours of storage. (g,h) Corresponding images with an additional spotted glycerol-water droplet (pos. 4) after a repeated cleaning procedure; time period between the delivery of droplets 3 and 4 about 43 min.

Large biological compounds such as proteins consist of many functional groups, which often lead to strong interactions with surfaces, making frontloading and cleaning procedures more challenging. Thus, the frontloading of AlexaFluor647-labeled secondary antibody IgG with a molecular weight of approx. 150 kDa into a micropipette and its subsequent cleaning was evaluated. The final concentration of the secondary antibody was 10 μg/mL, which corresponded to the manufacturer's recommended concentration for immunofluorescence staining. Similar to rhodamine 6G, the buffer solution of the antibody was mixed with 20 wt% glycerol. Previous studies have already shown that glycerol-buffer mixtures can be used as a solvent for fluorescent proteins without any denaturation effects, structural changes or activity loss [2,22]. The loading procedure was similar to the rhodamine 6G experiment, and the results are shown in Fig 5a–5d as bright-field images and corresponding fluorescence images. At first, a droplet consisting of the dye-labeled antibody was delivered onto the glass surface and its fluorescence signal could be observed (Fig 5a and 5b, pos. 1).

Then, the cleaning procedure was performed as indicated for the rhodamine 6G experiments, but this time the microchannel of the micropipette was cleaned with a 4% NaOCl solution instead of a 5% ethanol-water solution. Fig 5c and 5d shows the delivered antibody-containing droplet (pos. 1) as well as the delivered glycerol-water droplet after this cleaning procedure (pos. 2). Indeed, after performing the cleaning procedure, no residual fluorescence could be observed in the glycerol-water droplet (Fig 5d). However, the fluorescence signal from the antibody-containing droplet also disappeared (pos. 1 in Fig 5b *vs*. Fig 5d). The drying-out of the droplet could lead to changed fluorescence properties of AlexaFluor647, as already hypothesized for rhodamine 6G (see Fig 4b, pos. 2 *vs* pos. 4). In order to confirm this assumption, a 60-minute drying experiment in air was performed similar to that for rhodamine 6G (S2 Fig). Again, a time-dependent decrease in the fluorescence intensity was observed.

For storage over a few hours or several days, the cleaned and emptied (thus, only air-filled) AFM micropipettes were placed upside-down in small containers (e.g. multiwell plates). The containers were filled with bidistilled water just below the micropipette reservoir opening and were sealed with a parafilm sheet. The advantage of this storage method was the prevention of the micropipette opening drying-out, so that the micropipette could be used promptly and repeatedly in succession. The repeated usage of an AFM micropipette after several hours of storage in a water-filled multiwall plate is shown in Fig 5e–5h. At first, the stored micropipette was extricated from bidistilled water, which had entered the tip opening during the storage period, by applying a high-pressure pulse. Then, it was loaded again with the dye-labeled antibody and a new fluorescent droplet could be successfully delivered on a glass coverslip (Fig 5e and 5f, pos. 3). A subsequently delivered non-fluorescent water droplet indicated the successful cleaning procedure (Fig 5g and 5h, pos. 4). However, the fluorescence signal of the antibody-containing droplet also disappeared, as shown previously.

### Conclusions

In summary, we have introduced a rapid, versatile and cost-efficient frontloading and cleaning procedure for the repeated use of the same pressure-controlled microchanneled AFM micropipette. The proof of principle of the procedure could be demonstrated for an organic fluorescent dye and a protein solution. However, the frontloading procedure is also applicable for other complex compounds, such as printer’s ink or colloidal particles/nanoparticles. It also provides a simplified and faster handling for already known experiments with FluidFM. For instance, FluidFM has previously been described as a scanning probe lithography tool for metallic interactions. Here, it was used for the local fabrication of conductive metallic wires in liquid [23]. Through the combination of this nanolithography with the frontloading procedure, conductive layers with different compositions and sizes could be produced with only one micropipette in shortest time. Furthermore, the frontloading procedure could also be used as an adaptive method for the local manipulation of living cells with substances of very limited stability. For this reason, the frontloading procedure could be used as an advanced method for local polymer replacement for neuron patterning [24] or for local cell manipulation of secretory active epithelial tissues with biogenic amines or inhibitors of transport proteins [25,26].

In addition to the droplet deposition in air as demonstrated here, the frontloading and cleaning procedures can also be applied to experiments in a liquid environment. For the frontloading procedure, one would apply liquid volumes as small as possible in order to reduce any contamination at the micropipette outside. The external cleaning procedure would then require longer incubation times and/or more cleaning cycles to minimize residual molecules at the micropipette outside. However, depending on the target substance and the environmental complexity, the external cleaning procedure would need to be adjusted carefully. For instance, in a preliminary experiment, a rhodamine 6G-filled micropipette was immersed into a large water drop simulating a liquid environment. Here, a decrease in the environmental fluorescence signal could be observed when increasing the micropipette's external cleaning time (S3 Fig). Moreover, the combination of fluorescence microscopy and FluidFM is favorable for the application of fluorescent substances. When using a non-fluorescent substance, at first one would optimize frontloading and cleaning experiments with a fluorescent reference substance, which is similar to the target substance in terms of molecular weight, specific functional groups and solubility. However, these topics will be part of further studies.

## Supporting Information

S1 Fig Time series for the drying of a rhodamine 6G droplet in air. (a) Bright-field image of a freshly spotted droplet of 50 μM rhodamine 6G onto a glass coverslip and (b-f) corresponding fluorescence images at five different recording time points; scale bar = 50 μm; λ_ex_ = 485 nm ± 15 nm, λ_em_ = 535 nm ± 25 nm. (g) Bright-field image of the same droplet after drying for 60 min. (h) Fluorescence intensity profile plots of the droplet at the five different time points from (b-f) along the red line indicated in (a). (i) Time-dependent decrease in the fluorescence intensity in the region of the droplet within 60 min; image acquisition rate was 1 min^-1^.
(PDF)

S2 Fig Time series for the drying of a dye-labelled antibody droplet in air. (a) Bright-field image of a freshly spotted droplet of AlexaFluor647-labeled antibody IgG (10 μg/mL) onto a glass coverslip and (b-f) corresponding fluorescence images at five different recording time points; scale bar = 50 μm; λ_ex_ = 640 nm ± 15 nm, λ_em_ = 700 nm ± 37.5 nm. (g) Bright-field image of the same droplet after drying for 60 min. (h) Fluorescence intensity profile plots of the droplet at the five different time points from (b-f) along the red line indicated in (a). (i) Time-dependent decrease in the fluorescence intensity in the region of the droplet within 60 min; image acquisition rate was 1 min^-1^.
(PDF)

S3 Fig Immersion of a micropipette into a water drop after frontloading with rhodamine 6G and different cleaning cycles. The micropipette was filled with 50 μM rhodamine 6G *via* frontloading procedure by applying a droplet of approx. 5 μL onto the glass coverslip surface and dipping the micropipette into this droplet. Then, the micropipette was cleaned externally in four different cleaning cycles. Here, the NaOCl incubation time varies between 1 min and 4 min. Subsequently, the micropipette was also cleaned with 5% ethanol-water solution as long as the total cleaning procedure took about 5 min. After this, the micropipette was immersed into a previously delivered water drop for approx. 5 min. Then, the residual fluorescence signal within the water drop mainly resulting from any outside contamination was recorded in comparison to the background signal without any micropipette contact (bar “Water”). After four cleaning cycle tests, a small volume of rhodamine 6G was delivered from the micropipette into the water drop (bar “Rhodamine 6G”). λ_ex_ = 485 nm ± 15 nm, λ_em_ = 535 nm ± 25 nm.
(PDF)
