# Supplementary material for: A Multifunctional Frontloading Approach for Repeated Recycling of a Pressure-Controlled AFM Micropipette
Source: PLoS One. 2015 Dec 4;10(12):e0144157. doi: 10.1371/journal.pone.0144157 (PMC4670200; doi:10.1371/journal.pone.0144157)
Supplement: S1 Fig — (a) Bright-field image of a freshly spotted droplet of 50 μM rhodamine 6G onto a glass coverslip and (b-f) corresponding fluorescence images at five different recording time points; scale bar = 50 μm; λex = 485 nm ± 15 nm, λem = 535 nm ± 25 nm. (g) Bright-field image of the same droplet after drying for 60 min. (h) Fluorescence intensity profile plots of the droplet at the five different time points from (b-f) along the red line indicated in (a). (i) Time-dependent decrease in the fluorescence intensity in the region of the droplet within 60 min; image acquisition rate was 1 min-1. (PDF) [file pone.0144157.s001.pdf]

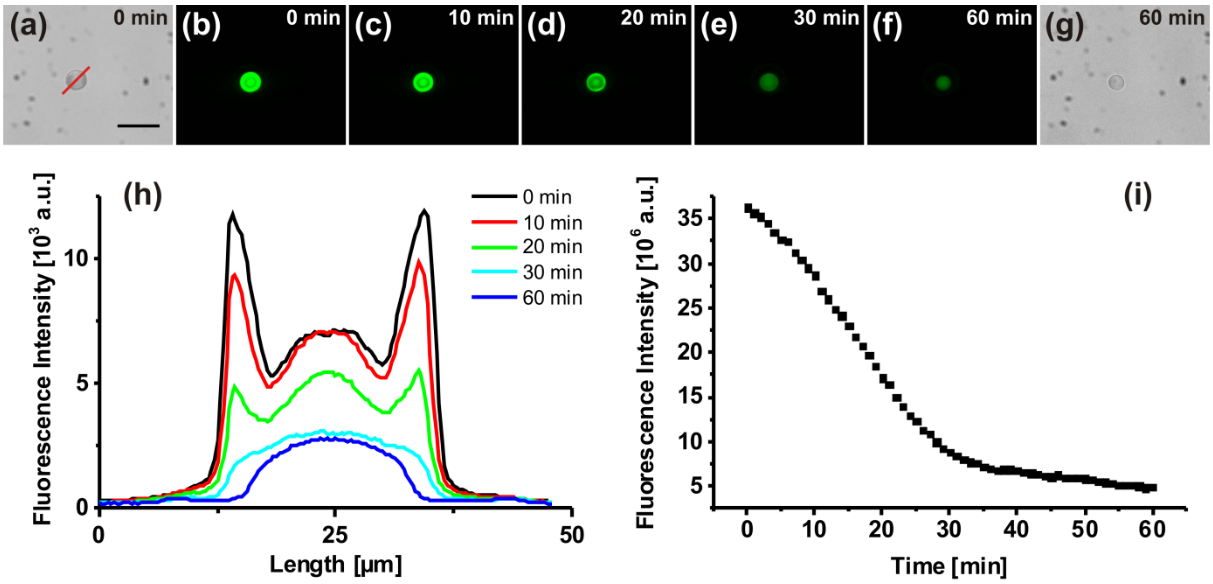

S1 Fig. Time series for the drying of a rhodamine 6G droplet in air.

(a) Bright-field image of a freshly spotted droplet of 50 µM rhodamine 6G onto a coverslip and (b-f) corresponding fluorescence images at five different recording time points; scale bar = 50 µm; λ_{ex} = 485 nm ± 15 nm, λ_{em} = 535 nm ± 25 nm. (g) Bright-field image of the same droplet after drying for 60 min. (h) Fluorescence intensity profile plots of the droplet at the five different time points from (b-f) along the red line indicated in (a). (i) Time-dependent decrease in the fluorescence intensity in the region of the droplet within 60 min; image acquisition rate was 1 min^{-1}.
