# Supplementary material for: A Multifunctional Frontloading Approach for Repeated Recycling of a Pressure-Controlled AFM Micropipette
Source: PLoS One. 2015 Dec 4;10(12):e0144157. doi: 10.1371/journal.pone.0144157 (PMC4670200; doi:10.1371/journal.pone.0144157)
Supplement: S3 Fig — The micropipette was filled with 50 μM rhodamine 6G via frontloading procedure by applying a droplet of approx. 5 μL onto the glass coverslip surface and dipping the micropipette into this droplet. Then, the micropipette was cleaned externally in four different cleaning cycles. Here, the NaOCl incubation time varies between 1 min and 4 min. Subsequently, the micropipette was also cleaned with 5% ethanol-water solution as long as the total cleaning procedure took about 5 min. After this, the micropipette was immersed into a previously delivered water drop for approx. 5 min. Then, the residual fluorescence signal within the water drop mainly resulting from any outside contamination was recorded in comparison to the background signal without any micropipette contact (bar “Water”). After four cleaning cycle tests, a small volume of rhodamine 6G was delivered from the micropipette into the water drop (bar “Rhodamine 6G”). λex = 485 nm ± 15 nm, λem = 535 nm ± 25 nm. (PDF) [file pone.0144157.s003.pdf]

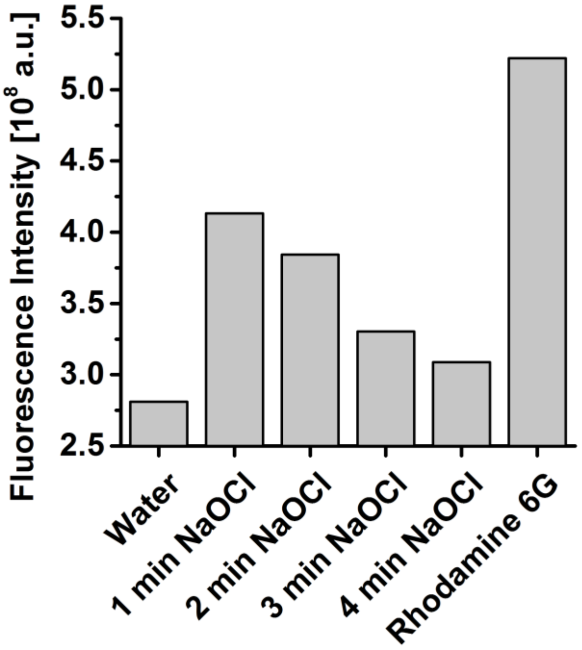

S3 Fig. Immersion of a micropipette into a water drop after frontloading with rhodamine 6G and different cleaning cycles.

The micropipette was filled with 50 µM rhodamine 6G *via* frontloading procedure by applying a droplet of approx. 5 µL on the glass coverslip surface and dipping the micropipette into this droplet. Then, the micropipette was cleaned externally within four different cleaning cycles. Here, the NaOCl incubation time varies between 1 min and 4 min. Subsequently, the micropipette was also cleaned with 5% ethanol-water solution as long as the total cleaning procedure accounted for 5 min. After this, the micropipette was immersed into a previously delivered water drop for approx. 5 min. Then, the residual fluorescence signal within the water drop mainly resulting from any outer contamination was recorded in comparison to the background signal without any micropipette contact (bar "Water"). After four cleaning cycle tests, a small volume of rhodamine 6G was delivered from the micropipette into the water drop (bar "Rhodamine 6G"). λ_{ex} = 485 nm ± 15 nm, λ_{em} = 535 nm ± 25 nm.
